# LncRNA H19 promotes odontoblastic differentiation of human dental pulp stem cells by regulating miR-140-5p and BMP-2/FGF9

**DOI:** 10.1186/s13287-020-01698-4

**Published:** 2020-05-27

**Authors:** Jialin Zhong, Xinran Tu, Yuanyuan Kong, Liyang Guo, Baishun Li, Wenchao Zhong, Ying Cheng, Yiguo Jiang, Qianzhou Jiang

**Affiliations:** 1 Key Laboratory of Oral Medicine, Guangzhou Institute of Oral Disease, Stomatology Hospital of Guangzhou Medical University, Huangsha Avenue 39, Guangzhou, 510000 People’s Republic of China; 2 State Key Laboratory of Respiratory Disease, Institute for Chemical Carcinogenesis, Guangzhou Medical University, Xinzao, Panyu District, Guangzhou, 511436 People’s Republic of China

**Keywords:** Long non-coding RNA, H19, Human dental pulp stem cells (hDPSCs), Dentinogenesis, Odontoblastic differentiation, miR-140-5p

## Abstract

**Background:**

Increasing evidence has revealed that long non-coding RNAs (lncRNAs) exert critical roles in biological mineralization. As a critical process for dentin formation, odontoblastic differentiation is regulated by complex signaling networks. The present study aimed to investigate the biological role and regulatory mechanisms of lncRNA-H19 (H19) in regulating the odontoblastic differentiation of human dental pulp stem cells (hDPSCs).

**Methods:**

We performed lncRNA microarray assay to reveal the expression patterns of lncRNAs involved in odontoblastic differentiation. H19 was identified and verified as a critical factor by qRT-PCR. The gain- and loss-of-function studies were performed to investigate the biological role of H19 in regulating odontoblastic differentiation of hDPSCs in vitro and in vivo. Odontoblastic differentiation was evaluated through qRT-PCR, Western blot, and Alizarin Red S staining. Bioinformatics analysis identified that H19 could directly interact with miR-140-5p, which was further verified by luciferase reporter assay. After overexpression of miR-140-5p in hDPSCs, odontoblastic differentiation was determined. Moreover, the potential target genes of miR-140-5p were investigated and the biological functions of BMP-2 and FGF9 in hDPSCs were verified. Co-transfection experiments were conducted to validate miR-140-5p was involved in H19-mediated odontoblastic differentiation in hDPSCs.

**Results:**

The expression of H19 was significantly upregulated in hDPSCs undergoing odontoblastic differentiation. Overexpression of H19 stimulated odontoblastic differentiation in vitro and in vivo, whereas downregulation of H19 revealed the opposite effect. H19 binds directly to miR-140-5p and overexpression of miR-140-5p inhibited odontoblastic differentiation of hDPSCs. H19 acted as a miR-140-5p sponge, resulting in regulated the expression of BMP-2 and FGF9. Overexpression of H19 abrogated the inhibitory effect of miR-140-5p on odontoblastic differentiation.

**Conclusion:**

Our data revealed that H19 plays a positive regulatory role in odontoblastic differentiation of hDPSCs through miR-140-5p/BMP-2/FGF9 axis, suggesting that H19 may be a stimulatory regulator of odontogenesis.

## Background

Due to the strong self-renewal capacity and regenerative properties, the biological effects of mesenchymal stem cells (MSCs) have been widely acknowledged and their application on bone tissue engineering have made great progress [1]. As a promising type of adult stem cell, human dental pulp stem cells (hDPSCs) can proliferate and differentiate into odontoblasts and subsequently deposit reparative dentine as a stress reaction when the pulp tissue is subject to bacterial, chemical, or physical stimulation [2]. Owing to the enormous proliferative capacity and multi-potentiality, hDPSCs are capable for adipogenic, osteogenic, dentinogenic, and neurogenic differentiation under specific induction environments [3]. Thus, the complex molecular mechanisms that promote odontogenic differentiation of hDPSCs are essential for further investigation.

Long non-coding RNAs (lncRNAs) play crucial roles in numerous physiological and pathological activities in cells. A variety of functions of lncRNAs were found in recent years in embryonic development, inflammation, cell differentiation, and tumors [4–6]. Recent studies have shown that lncRNAs can regulate the differentiation of stem cells [7–9]. Several functions of lncRNAs in biological mineralization have been elucidated [10]. However, few studies have focused on the role of lncRNAs and their molecular mechanisms underlying odontoblastic differentiation of hDPSCs.

In this study, lncRNA microarray results revealed that lncRNA-H19 (H19) was significantly upregulated in hDPSCs undergoing odontoblastic differentiation. We investigated the function role of H19 in the odontoblastic differentiation of hDPSCs. Overexpression of H19 notably promoted odontogenic differentiation of hDPSCs in vitro and in vivo. In accordance with the bioinformatics analysis, the mineralization-related miR-140-5p which had potential binding sites with H19 was selected for further study. We demonstrated that H19 could accelerate odontogenic differentiation of hDPSCs via binding miR-140-5p to upregulate bone morphogenetic protein-2 (BMP-2) and fibroblast growth factor 9 (FGF9) expression. Therefore, this research may thus provide a novel regulatory mechanism of lncRNAs in odontogenic differentiation.

## Materials and methods

### Cell culture

All DPSCs in our experiments derived from human patients. Cells were isolated from the extracted third molars of human dental patients between 18 and 22 years old. The process was reviewed and approved by the Institutional Review Board of the Hospital of Stomatology of Guangzhou Medical University, and a signed informed consent document was obtained from each patient. hDPSCs were prepared using explants and the trypsin digestion technique. Cells were cultured and expanded in alpha minimum essential medium (α-MEM) supplemented with 10% (v/v) fetal bovine serum (FBS) and 1% (v/v) penicillin-streptomycin solution. Cells were maintained in a humidified atmosphere of 5% CO_2_ at 37 °C. Fresh culture media was replenished every 3 days. Third-passage cells were harvested for the subsequent experiments.

### Flow cytometry assay

Cultured cells in their 3rd passage were identified based on the surface antigens of hDPSCs using a flow cytometry method. Cells were trypsinized and incubated in PBS containing 0.1% FBS for 45 min with fluorescein-conjugated monoclonal antibodies against CD34, CD45, CD73, CD90, CD146, and CD166 (BD Biosciences, San Jose, CA, USA). Flow cytometry analysis was performed using a flow cytometer (FACSCalibur, BD Biosciences).

### LncRNA microarray

After odotoblastic induction for 3 days, the differentiated hDPSCs in the culture dishes were isolated and hDPSCs cultured in normal culture medium were observed as the control. Total RNA was isolated with TRIzol (Invitrogen) according to the manufacturer’s instructions. A lncRNA microarray (KangChen Bio-tech, Shanghai, China) using induced group and non-induced group was performed. Differentially expressed lncRNAs were identified based on fold change (> 2), as well as *P* < 0.05.

### Odontogenic differentiation induction and treatment of hDPSCs

hDPSCs (passages 4~6) were seeded in 6-well plates at a density of 1 × 10^5^ cells each well. To induce odontogenic differentiation of hDPSCs, cells were cultured with odontogenic medium (10% FBS, 10^−8^ mol/L dexamethasone, 10 mmol/L β-glycerophosphate, and 50 mg/mL ascorbic acid in α-MEM) for 14 days. Control samples were cultured in 10% FBS in α-MEM with no supplements. For functional experiments, recombinant human protein BMP-2 (rhBMP-2; Gibco, USA) and FGF9 (rhFGF9; Peprotect, USA) were purchased and used to stimulate hDPSCs. hDPSCs were cultured with odontogenic medium consisting of rhBMP-2 (20 ng/mL) and rhFGF9 (20 ng/mL) separately. Fresh medium was changed every 3 days.

### ALP assay and Alizarin Red S staining

Cell lysates were harvested after odontoblatic differentiation, and ALP activity was evaluated and analyzed following the manufacturer’s instructions (ALP Diagnostics Kit, Yeasen, Shanghai, China). hDPSCs were fixed in 4% paraformaldehyde, and mineral nodules were detected by Alizarin Red S staining (BestBio, Shanghai, China).

### RNA preparation and qRT-PCR

Total RNA was isolated from cultured cells with TRIzol Reagent (Invitrogen, USA) according to the manufacturer’s protocol. Reverse transcription was performed with PrimeScript RT Master Mix (TaKaRa, Japan) for lncRNA and mRNA. For miRNA examination, cDNA was synthesized using a miRNA First Strand cDNA Synthesis Kit (Sangon Biotech, China). RNA expression was measured by quantitative real-time PCR analysis using a CFX96 Real-Time PCR instrument (Life Technology, USA) and SYBR Green Reagent (TaKaRa, Japan). GAPDH was used as an internal control for lncRNA and mRNA. The expression of miRNA was normalized to U6. Primers were synthesized by Generay Technologies (Shanghai, China), and all sequences are available in Additional file 1: Table 1. The 2^−ΔΔCT^ method was used to calculate relative expression levels.

### Western blot analysis

Cells were lysed in RIPA buffer (Beyotime, China) combined with a cocktail of protease inhibitors (Thermo Scientific, Rockford, IL). Equal amounts of proteins (25 μg) of different groups were separated by 10% SDS-PAGE and transferred to 0.45-μm PVDF membranes (Millipore, USA). The membrane was first blocked with 5% BSA for 1 h at room temperature and incubated at 4 °C overnight with primary antibodies: GAPDH (1:1000; Abcam, Cambridge, UK), RUNX2(1:1000; Cell Signaling Technology, Danvers, MA), DSPP (1:1000; Abcam, Cambridge, UK), and DMP-1 (1:1000; Abcam, Cambridge, UK). After washed with Tris-buffer saline containing 0.05% Tween 20 (TBST) for three times and 5 min each, the membranes were incubated with secondary antibodies labeled with horseradish peroxidase (1:3000; Cell Signaling Technology, Danvers, MA) at room temperature for 1 h. Blots were visualized using ECL Western Blotting Substrate.

### Cell transfection

Recombinant lentiviruses targeting H19 (shH19-1 and shH19-2) and Lenti-shNC were purchased from GenePharma Company (Shanghai, China). hDPSCs were transfected by lentiviruses exposure in 1 mL α-MEM supplemented with 10% FBS and 5 μg/mL polybrene for 24 h. H19 overexpression plasmid pcDNA3.1-H19, miR-140-5p mimics, and scramble control (NC) were chemically synthesized by GenePharma. When hDPSCs were 70–80% confluent, pcDNA-H19 and miRNA mimic transfection was performed using Lipofectamine 3000 (Invitrogen, USA) according to the manufacturer’s instructions. qRT-PCR analysis was used to detect H19 and miR-140-5p expression levels to validate the transfection efficiencies. The cells were cultured in mineralizing medium for odontoblastic differentiation 48 h after transfection.

### Dual luciferase reporter assay

The luciferase reporter vector psiCHECK-2 containing full length of H19 sequence, the 3′ untranslated region (UTR) sequence of BMP-2/FGF9, and their relevant mutant types were synthesized by Synbio, China. HEK293T cells were seeded into 24-well plates at a density of 5 × 10^4^ cells per well the day before transfection. Cells were transiently co-transfected with corresponding psiCHECK-2 vector and miR-NC/miR-140-5p mimics using Lipofectamine 3000. Renilla and Firefly luciferase activity were measured separately 48 h after transfection using a dual-luciferase reporter assay system (Promega, USA) following the manufacturer’s instructions. These experiments were repeated three times.

### ELISA assay

The human enzyme-linked immunosorbent assay (ELISA) kits were obtained from CLOUD-CLONE CORP (Wuhan, China). The culture supernatants were collected on day 2 after odontogenic induction treatment. The secreted levels of BMP-2 and FGF9 were quantified using corresponding ELISA kits, according to the manufacturer’s protocol.

### In vivo odontogenesis assay

BALB/c nude mice (5 weeks old, five mice were included in each group) were purchased from the Experimental Animal Center, Guangzhou University of Chinese Medicine. All the animal care and experimental procedures were approved by the Institutional Animal Care and Use Committee of Guangzhou University of Chinese Medicine and were performed in accordance with established guidelines. hDPSCs with H19 overexpression and negative controls induced under odontoblastic medium for 1 week were harvested and incubated with Bio-Oss Collagen (Geistlich, Germany) scaffolds for 1 h at 37 °C. The hDPSC-loaded scaffolds were implanted subcutaneously into the nude mice. Implants were harvested 6 weeks after implantation and fixed in 4% PFA.

### Analyses of bone formation in vivo

Micro-CT analysis was performed using a high-resolution micro-CT (Bruker, Karlsruhe, Germany) set as 10 μm of the voxel resolution of the scanned volumes, 500 ms of the 360 rotational steps per time. Micro-CT image slices were reconstructed, and the ratio of new bone volume to existing tissue volume (BV/TV) was calculated by micro-CT image analysis software. For histological examination, the specimens were decalcified in 10% EDTA (pH 7.4) for 30 days. After dehydrated and embedded with paraffin, tissue samples of 5-μm-thick paraffin section were stained with hematoxylin and eosin (H&E) and Masson’s trichrome. Immunohistochemical staining was conducted using HRP/DAB (ABC) detection IHC kit and human-specific primary antibodies, including DMP-1 (1:100). All products for IHC were from Abcam. Histological staining was captured under the microscope (Leica Microsystems, Germany) with an increase of × 100.

### Statistical analysis

Statistical analyses were performed using GraphPad Prism7.0 (GraphPad Prism, Inc., La Jolla, CA, USA). One-way analysis of variance (ANOVA) and Student’s *t* test (two-tailed) were used to evaluate the statistical significance. All data are shown as the means ± SD from three independent experiments. Statistical significance was defined as *P* < 0.05.

## Results

### Characteristics of hDPSCs derived from adult dental pulp

**Fig. 1:**
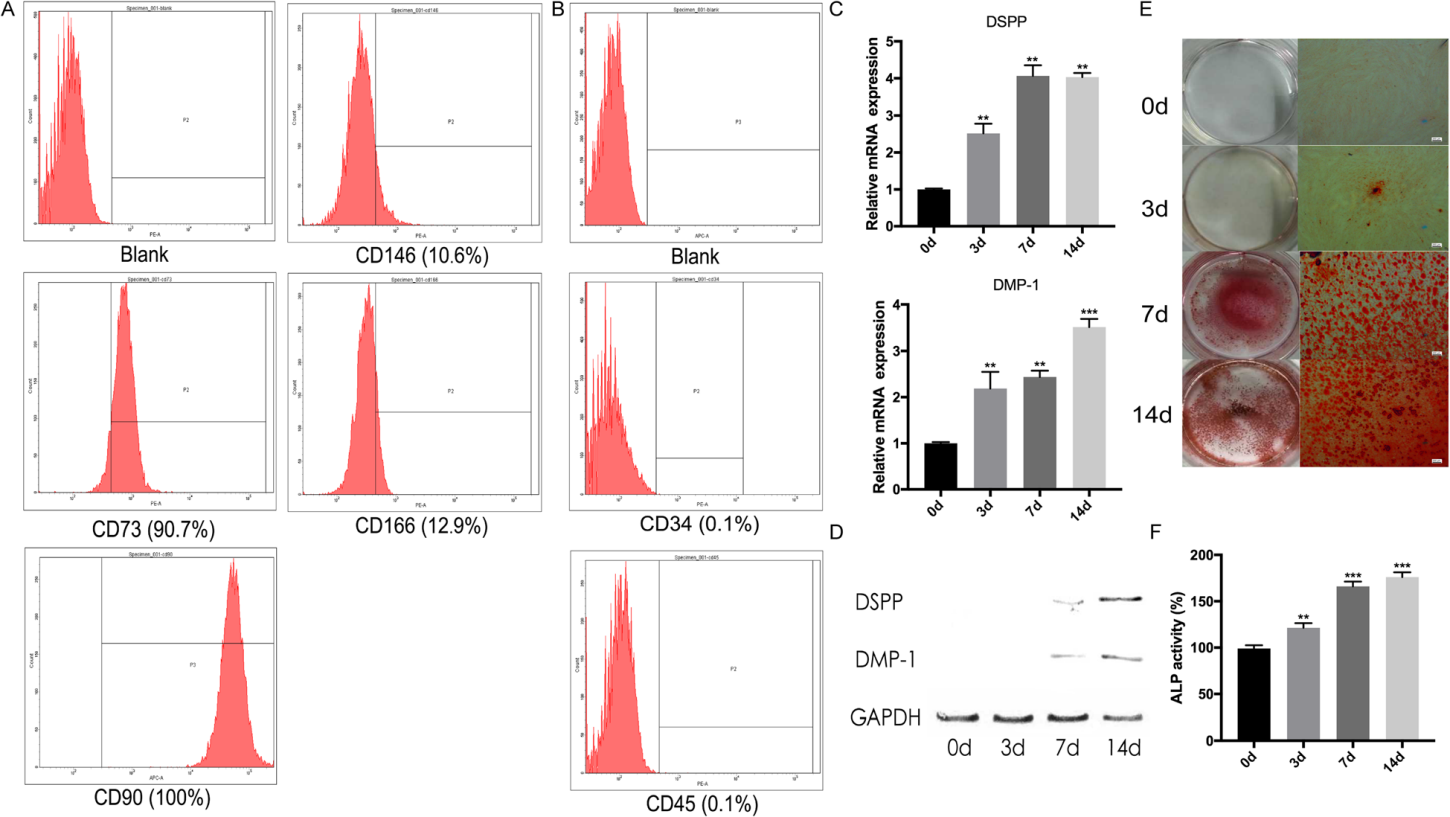
Flow cytometry of hDPSCs and odontoblastic differentiation of hDPSCs after induction for 14 days. **a** Representative diagrams are shown for the PE negative control, CD34, CD45, CD73, CD146, and CD166. **b** Representative diagrams are shown for the APC negative control and CD90. **c**, **d** The mRNA and protein expression levels of DMP-1 and DSPP increased during odontoblastic differentiation. **e** The number of mineralized nodules increased with the process of odontoblastic differentiation. **f** The ALP activity of hDPSCs was enhanced after differentiation induction. The data are presented as the mean ± SD in three independent experiments. *P < 0.05, **P < 0.01, ***P < 0.001

hDPSCs were identified with flow cytometry. hDPSCs exhibited high expression of CD73 (90.7%), CD90 (100%), CD146 (10.6%), and CD166 (12.9%) and were negative for CD34 (0.1%) and CD45 (0.1%) (Fig. 1a, b). These results indicated that hDPSCs highly expressed mesenchymal cell surface molecular markers and scarcely expressed hematopoietic system-derived cell surface markers. Furthermore, the differentiation capacities of hDPSCs were assessed. The mRNA expression levels of odontogenesis-related genes DSPP and DMP-1 were upregulated gradually during odontogenic differentiation (Fig. 1c). Western blot analysis revealed a similar trend that the protein levels of DSPP and DMP-1 were also enhanced significantly after odontogenic induction (Fig. 1d). Matrix mineralization and ALP activity were increased significantly in the process of odontogenic induction as compared to the normal culture group (Fig. 1e, f).

### Microarray expression profile analysis of lncRNAs in hDPSCs during differentiation induction

Whether lncRNAs involved in the odontoblastic differentiation of hDPSCs was verified by microarray. Compared with the normal culture group, 1106 lncRNAs were identified to significantly differentially expressed (fold change > 2.0; *P* < 0.05) after 3 days of odontoblastic induction in hDPSCs. Among these, 617 lncRNAs were upregulated, while 489 lncRNAs were downregulated (Additional file 1: Figure S1A). Among the significantly upregulated lncRNAs, mineralization-related H19, MALAT1, MIR31HG, and WNT2 were chosen as candidate lncRNAs. To prove the accuracy of the microarray results, qRT-PCR was used to investigate the expression level of four lncRNAs at each time point during differentiation induction. It revealed that H19 was significantly upregulated 5.9-fold after induction for 7 days (Additional file 1: Figure S1B–E). Therefore, we focused on H19 for further study.

### H19 promotes the odontoblastic differentiation of hDPSCs

**Fig. 2:**
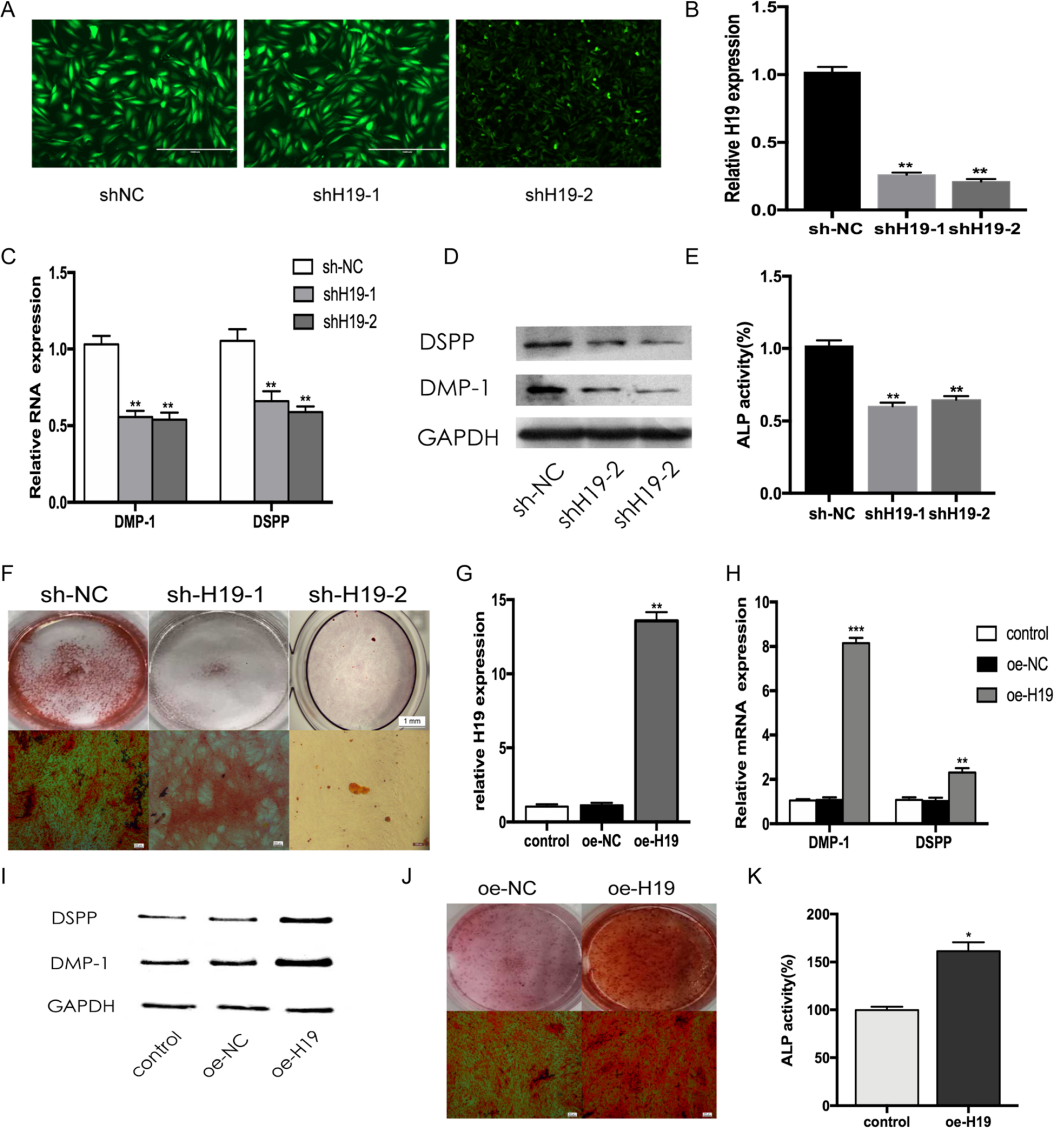
H19 promotes odontogenesis of hDPSCs. **a** Fluorescence was observed under an inverted fluorescence microscope after transfection for 48 h. **b** The expression levels of H19 were determined by qRT-PCR. **c** Relative mRNA expressions of DMP-1 and DSPP measured by qRT-PCR after odontoblast induction for 7 days. **d** Western blot results showed the protein levels of DSPP and DMP-1 decreased in shH19-1 and shH19-2 groups after odontoblast induction for 7 days. **e** The ALP activity was decreased by H19 knockdown after odontoblast induction for 7 days. **f** Images of Alizarin Red S staining in sh-NC, shH19-1, and shH19-2 groups. hDPSCs were cultured in odontoblastic medium for 7 days. **g** H19 was upregulated after hDPSCs were transfected with pcDNA 3.1-H19. **h** qRT-PCR assay revealed that the mRNA expressions of DMP-1 and DSPP were increased in H19 overexpression group after odontoblast induction for 7 days. **i** Western blot analysis of protein expression of DMP-1 and DSPP in hDPSCs after odontoblast induction for 7 days. **j** After odontoblast induction for 7 days, Alizarin Red S staining showed that H19 overexpression group generated more calcified nodules than control group. **k** The ALP activity was increased by H19 overexpression after odontoblast induction for 7 days. The data are presented as the mean ± SD in three independent experiments. *P < 0.05, **P < 0.01, ***P < 0.001

To investigate the function of H19, we stably silenced H19 expression in hDPSCs with lentiviruses. The transfection effects were observed under an inverted fluorescence microscope. Enhanced green fluorescent protein (EGFP) showed that hDPSCs were infected with the lentiviruses (Fig. 2a). qRT-PCR indicated that the expression level of H19 was downregulated by approximately 74.3% in shH19-1 group and 79.3% in shH19-2 group compared with that of the sh-NC group (*P* < 0.01 vs control group) (Fig. 2b). After odontoblastic induction for 14 days, downregulation of H19 resulted in significantly inhibited odontoblastic differentiation, characterized by lower expression levels of DSPP and DMP-1, weaker ALP activity, and fewer mineralization nodules (Fig. 2c–f). Correspondingly, the expression level of H19 was increased approximately 13-fold after transfected with overexpression plasmid (Fig. 2g). Consistent with the results above, the forced expression of H19 led to a stronger hDPSC capacity to differentiate into odontoblasts during odontoblastic induction for 14 days (Fig. 2h–k).

### H19 facilitates the odontogenesis of hDPSCs in vivo

**Fig. 3:**
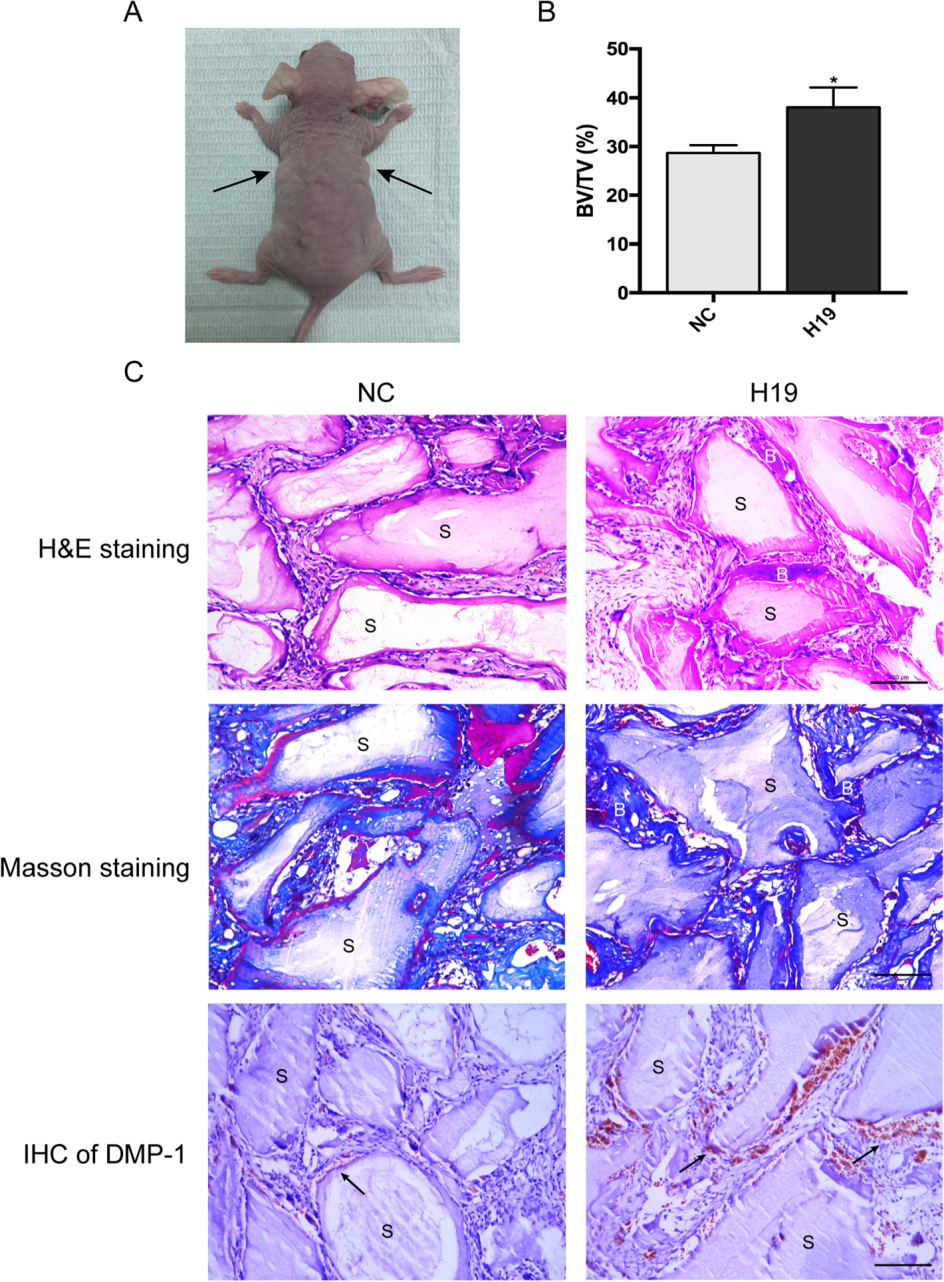
H19 enhances the dentinogenesis of hDPSCs in vivo*.***a** hDPSCs in NC and H19 group were transplanted subcutaneously into 5-week-old BALB/c homozygous nude mice for 6 weeks. **b** Percentages of new bone volume to tissue volume (BV/TV) of hDPSCs-loaded scaffolds. The data are presented as the mean ± SD of three experiments. *P < 0.05. **c** H&E staining, Masson staining, and immunohistochemical staining of DMP-1 in NC and H19 groups. B: dentin-like tissues, S: scaffold, scale bar = 100 μm

To further confirm the role of H19 in odontoblastic differentiation of hDPSCs, the in vivo experiments were conducted. After induction culture for 1 week in vitro, the H19 overexpression and negative control transfected hDPSCs were loaded on Bio-Oss Collagen scaffolds. The seeded scaffolds were then gently implanted subcutaneously into BALB/c nude mice (five mice per group) for 6 weeks (Fig. 3a). Three-dimensional reconstructed micro-CT analysis revealed the BV/TV increased significantly in H19-overexpressing group compared with the control group (Fig. 3b). The histological observations revealed a similar trend of BV/TV. H&E staining indicated more dentin-like structure formation around the area of scaffolds in H19-overexpressing group. In addition, more blue-colored bone collagen deposit was observed by Masson staining in H19-overexpression group, compared with the control group. Moreover, IHC analysis was applied to detect the protein expression of DMP-1 in specimens of each group. The abundance of DMP-1 was increased obviously in H19-overexpressing group, suggesting that upregulation of H19 could promote odontogenic differentiation of hDPSCs in vivo (Fig. 3c).

### H19 serves as a miRNA sponge for miR-140-5p in hDPSCs

**Fig. 4:**
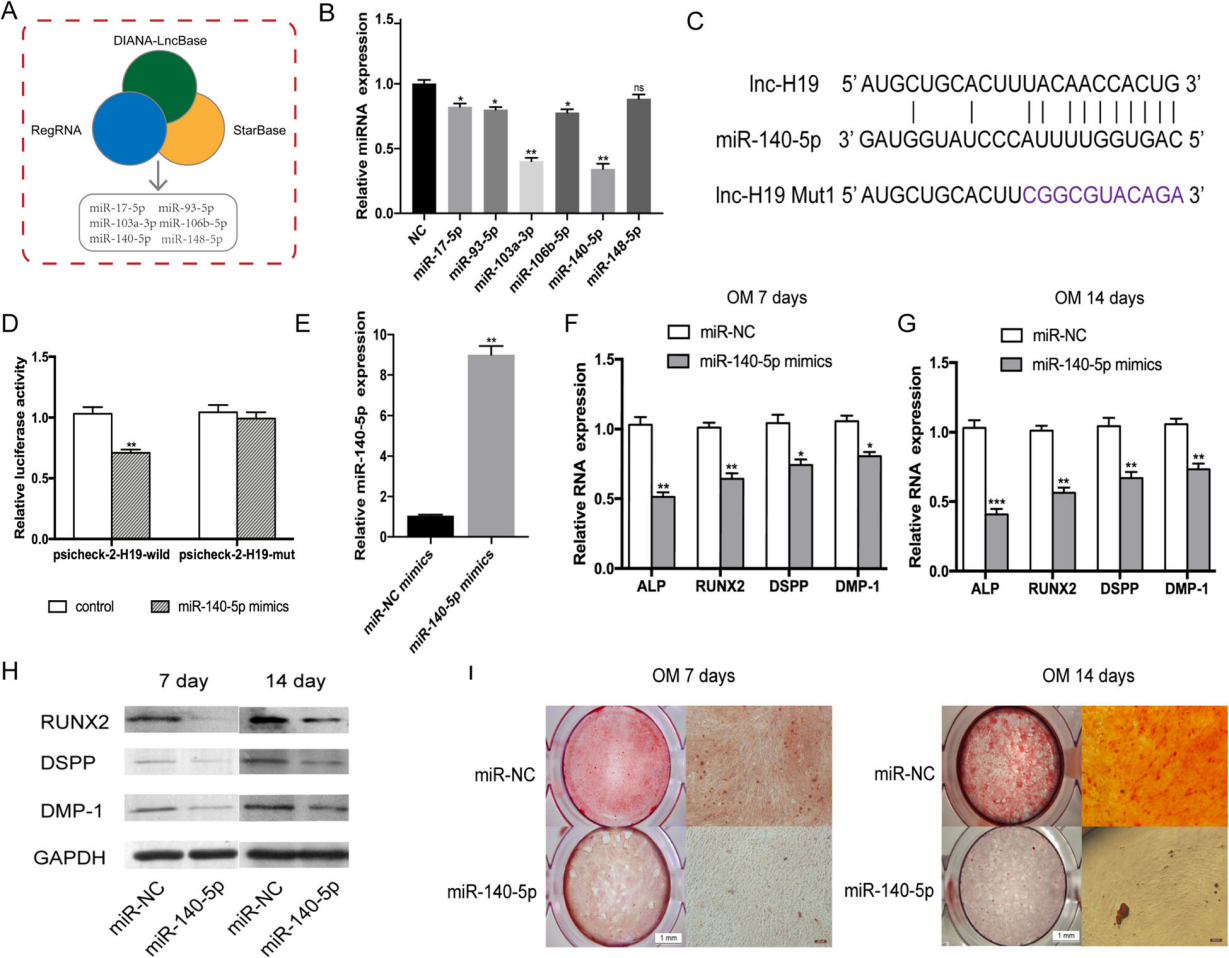
H19 binds directly to miR-140-5p and overexpression of miR-140-5p inhibits odontoblastic differentiation of hDPSCs. **a** Databases consistently predicted six miRNAs interacting with lncRNA H19. **b** Relative candidate miRNA expression after odontoblast induction for 14 days compared with that in NC groups. **c** Sequence alignment of H19 with wild-type and mutant-type miR-140-5p. The potential binding sites were predicted by Starbase. **d** Relative luciferase activity of HEK293T cells co-transfected with H19 reporter plasmid and miR-140-5p mimics. **e** qRT-PCR analysis was performed to measure the levels of miR-140-5p. **f** After odontoblast induction for 7 days, relative mRNA expressions of ALP, RUNX2, DSPP, and DMP-1 in miR-140-5p mimic group were measured by qRT-PCR. **g** Relative mRNA expressions of ALP, RUNX2, DSPP, and DMP-1 in miR-140-5p mimic groups and miR-140-5p mimic groups after odontoblast induction for 14 days. **h** Relative protein expression of RUNX2, DSPP, and DMP-1 in miR-140-5p mimic transfection groups after odontoblast induction for 7 days and 14 days. **i** Representative micrographs of Alizarin Red S staining in miR-140-5p mimic transfection groups after odontoblast induction for 7 days and 14 days. The data are presented as the mean ± SD in three independent experiments. *P < 0.05, **P < 0.01, ***P < 0.001

Computational analysis was performed using Starbase (http://starbase.sysu.edu.cn), DIANA-LncBase (http://www.microrna.gr/LncBase), and RegRNA (http://regrna2.mbc.nctu.edu.tw) databases, and six collectively predicted miRNAs (miR-17-5p, miR-93-5p, miR-103a-3p, miR-106b-5p, miR-140-5p, and miR-148-5p) may act as biological targets of H19 (Fig. 4a). The expression patterns of the candidate miRNAs in hDPSCs during odontoblastic differentiation were detected by qRT-PCR. Impressively, the expression levels of miR-140-5p were significantly decreased compared with those of the non-induction group (Fig. 4b). In addition, we conducted a dual-luciferase reporter assay to validate the interaction between H19 and miRNAs. The results revealed that miR-140-5p significantly reduced the luciferase activity of H19-wild compared to that of H19-mut, indicating that H19 might function as a sponge of miR-140-5p (Fig. 4c, d).

### Overexpression of miR-140-5p inhibits odontoblastic differentiation

To investigate whether miR-140-5p plays a role in regulating odontoblastic differentiation, miR-140-5p mimics were transfected into hDPSCs and the efficiency was evaluated 48 h after transfection. The qRT-PCR results showed that miR-140-5p transfected hDPSCs had an 8.9-fold higher miR-140-5p expression compared with miR-NC mimics transfected hDPSCs (Fig. 4e). Following odontoblastic induction for 7 and 14 days, relative mRNA expressions of ALP, RUNX2, DSPP, and DMP-1 were markedly suppressed in the miR-140-5p overexpression group as compared to the mimic NC group (Fig. 4f, g). Consistently, Western blot assay revealed that the odontogenic markers RUNX2, DSPP and DMP-1 were greatly decreased in miR-140-5p overexpression group (Fig. 4h). In addition, after odontoblastic induction for 7 days, the matrix mineralization level was remarkably decreased in miR-140-5p overexpression group assessed by Alizarin Red S staining. Overexpression of miR-140-5p continued to decrease calcified nodules after induction culture for 14 days (Fig. 4i). Briefly, these results indicated that miR-140-5p could suppress the odontoblastic differentiation of hDPSCs.

### H19 regulates BMP-2/FGF9 expression by binding to miR-140-5p

**Fig. 5:**
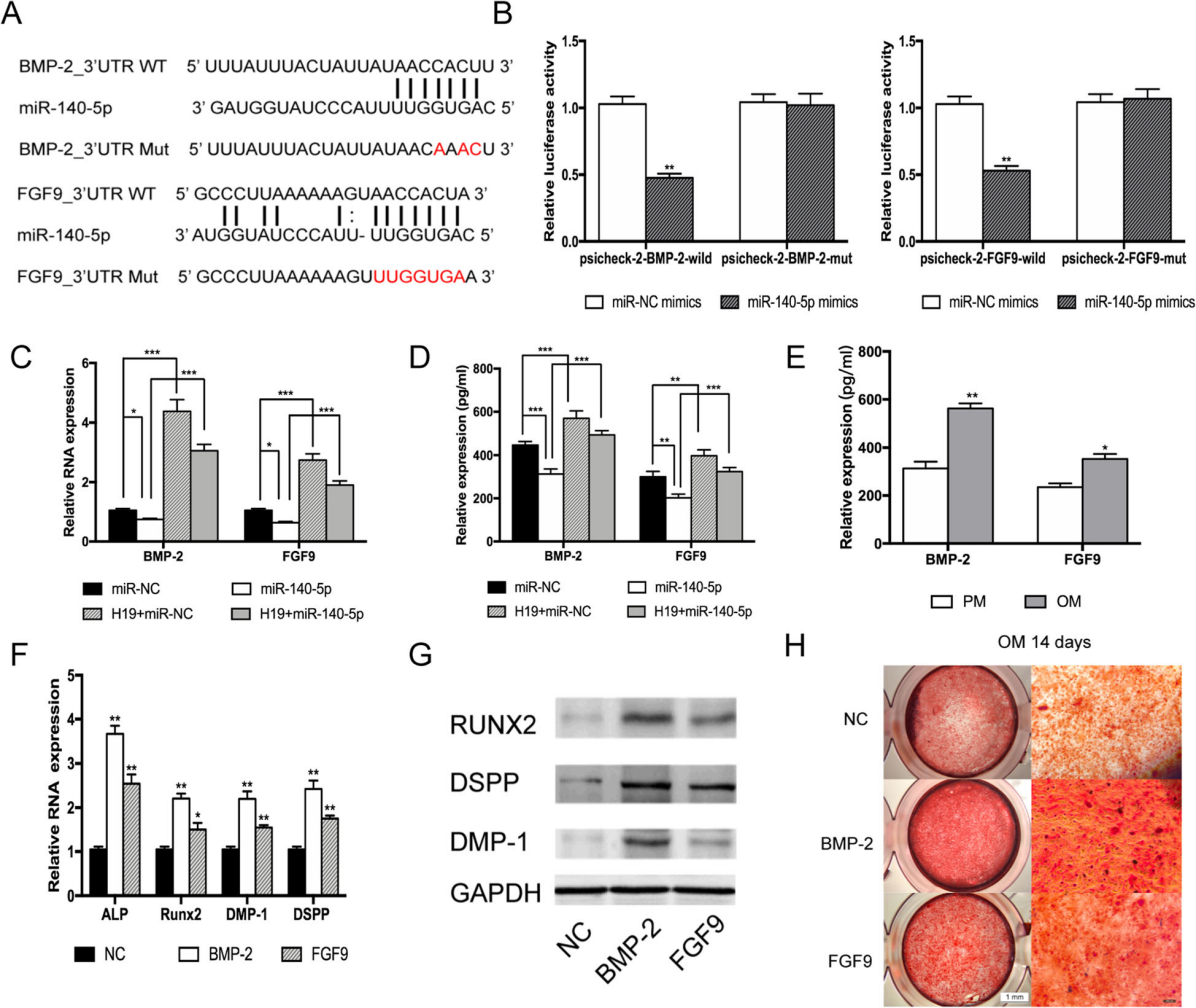
H19 regulates BMP-2/FGF9 expression by binding to miR-140-5p. **a** Schematic of BMP-2/FGF9 wild-type (WT) and mutant (Mut) luciferase reporter vectors. **b** miR-140-5p significantly reduced luciferase activity in BMP-2/FGF9-wild not in BMP-2/FGF9-mut. **c** Quantification of mRNA expression of BMP-2 and FGF9 measured by qRT-PCR in miR-140-5p mimics, pcDNA3.1-H19, miR-140-5p mimics/pcDNA3.1-H19 co-transfected hDPSCs relative to the control groups. **d** Relative expression of BMP-2 and FGF9 in hDPSCs measured by ELISA in miR-140-5p mimics, pcDNA3.1-H19, and miR-140-5p mimics/pcDNA3.1-H19 co-transfected hDPSCs. **e** Relative expression of BMP-2 and FGF9 in hDPSCs measured by ELISA after odontoblast induction for 14 days. **f** Relative mRNA expressions of ALP, RUNX2, DSPP, and DMP-1 in exogenous BMP-2-treated groups and exogenous FGF9-treated groups after odontoblast induction for 14 days. **g** Relative protein expressions of RUNX2, DSPP, and DMP-1 in exogenous BMP-2-treated groups and exogenous FGF9-treated groups after odontoblast induction for 14 days. **h** Representative micrographs of Alizarin Red S staining in exogenous BMP-2-treated groups and exogenous FGF9-treated groups after odontoblast induction for 14 days. The data are presented as the mean ± SD in three independent experiments. *P < 0.05, **P < 0.01, ***P < 0.001

Further prediction of target genes of miR-140-5p was performed by miRDB (http://mirdb.org) and TargetScan (http://www.targetscan.org/vert_72/). Notably, we found that the 3′ untranslated region (UTR) of the mineral-associated genes BMP-2 and FGF9 contained miR-140-5p binding sites (Fig. 5a). To confirm this finding, luciferase reporter assays were performed using psiCHECK-2 vector containing wild-type or mutant version of BMP-2/FGF9 3′ untranslated region (3′UTR) respectively. After co-transfected with miR-140-5p mimics and psiCHECK-2 vector for 48 h in HEK293T cells, the luciferase reporter results showed that obviously reduced luciferase activity was observed in BMP-2-wild not in BMP-2-mut. Meanwhile, the luciferase activity was remarkably reduced in HEK293T cells co-transfected with miR-140-5p mimics and FGF9-wt 3′UTR vector, but not significantly changed in HEK293T cells co-transfected with miR-140-5p mimics and the FGF9-mut 3′UTR vector (Fig. 5b). Furthermore, we conducted rescue assays to estimate whether BMP-2 and FGF9 act as downstream targets of H19/miR-140-5p by co-transfecting H19 and miR-140-5p mimics in hDPSCs. The RT-PCR analysis and ELISA assay results showed that transfection with miR-140-5p mimics inhibited the expression of BMP-2 and FGF9 in hDPSCs, while co-transfected with H19 could partly abolish this effect. The above findings confirmed BMP-2 and FGF9 are direct targets of miR-140-5p (Fig. 5c, d).

After elucidated that BMP-2 and FGF9 are direct targets of miR-140-5p, we next gain further insights into the biological influence of BMP-2 and FGF9 on hDPSCs. The ELISA assay results revealed the expressions of BMP-2 and FGF9 increased along with odontogenesis process (Fig. 5e). Since BMP-2 and FGF9 were secretory proteins, exogenous BMP-2 and FGF9 were utilized to stimulate hDPSCs. Odontogenic induction medium with 20 ng/mL recombinant BMP-2 or FGF9 was treated with hDPSCs separately for 14 days. Subsequent qRT-PCR assay and Western blot assay revealed that exogenous BMP-2/FGF9 facilitated the gene expression levels of odontogenic markers, ALP, Runx2, DSPP, and DMP-1, compared with the common odontogenic induction group (Fig. 5f, g). Meanwhile, the Alizarin Red S staining indicated that odontoblastic differentiation ability of hDPSCs treated with BMP-2/FGF9 was enhanced (Fig. 5h). Taken together, these data indicated that H19 acts upstream of miR-140-5p and inhibits the effect of miR-140-5p to target BMP-2/FGF9.

### H19 abrogates the inhibitory effect of miR-140-5p on odontoblastic differentiation

**Fig. 6:**
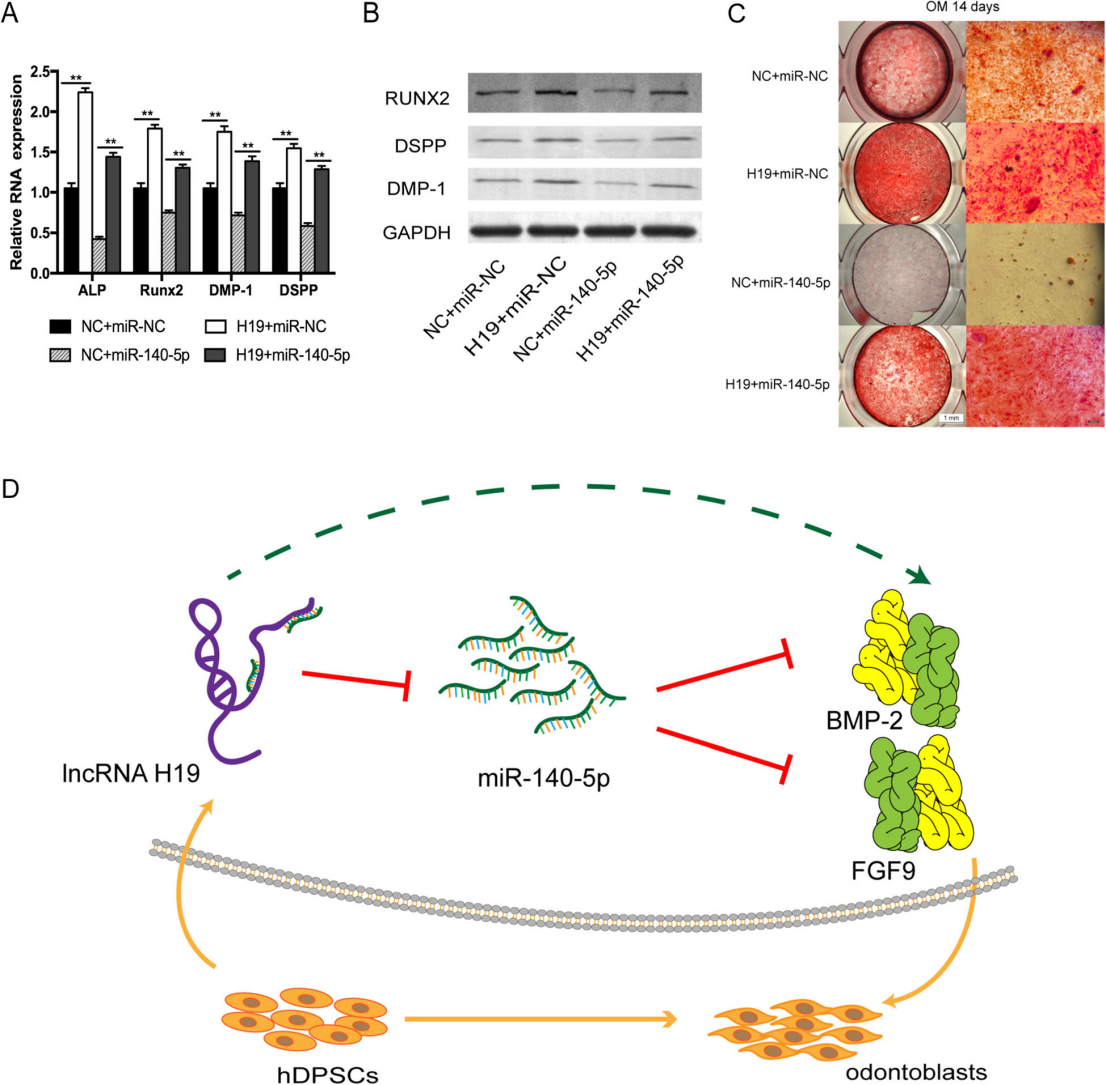
Overexpression of miR-140-5p reversed the stimulation effect of H19 in vitro. **a** Relative RNA expression of mineralization-related genes in the miR-140-5p co-transfection experiment after odontoblast induction for 14 days. **b** Relative protein expression of RUNX2, DSPP, and DMP-1 in the miR-140-5p co-transfection experiment after odontoblast induction for 14 days. **c** Images of Alizarin Red S staining in the miR-140-5p co-transfection experiment. hDPSCs were cultured in odontoblastic medium for 14 days. Histograms revealed quantification of Alizarin Red S staining by spectrophotometry. **d** The schematic diagram for lncRNA-H19/miR-140-5p/BMP-2/FGF9 axis. The data are presented as the mean ± SD in three independent experiments. *P < 0.05, **P < 0.01

Since H19 plays a ceRNA role by binding to miR-140-5p, rescue experiments were performed to validate whether miR-140-5p was involved in H19-mediated odontoblastic differentiation in hDPSCs. For the rescue assays, the H19 overexpression plasmid and miR-140-5p mimics were co-transfected into hDPSCs. The downregulated expression of odontoblast-related genes indicated that miR-140-5p mimics attenuated the promoting odontoblastic differentiation effect of H19 (Fig. 6a). Western blot assays revealed that the effects of miR-140-5p inhibition on the odontoblastic differentiation of hDPSCs could be significantly reversed by overexpressing H19 (Fig. 6b). Moreover, Alizarin Red S staining revealed that the overexpression of miR-140-5p overcame the promotion effects of increased H19 on odontoblastic differentiation (Fig. 6c). A summarized figure revealed the role and regulatory mechanisms of H19 in this study (Fig. 6d).

## Discussion

Human dental pulp stem cells (hDPSCs) were first isolated and named by Gronthos et al. [11]. The odontoblastic differentiation of hDPSCs is a crucial factor in reparative dentin generation and dental tissue self-repair in inflammatory microenvironments [12].

LncRNAs are involved in a variety of physiological and pathological activities of cells, including proliferation, differentiation, migration, and apoptosis [13–15]. Previous studies have revealed that lncRNAs play important roles in osteogenic differentiation of mesenchymal stem cells (MSCs) [16–19]. In this study, lncRNA microarray analysis using differentiation-induced and un-induced hDPSCs was performed to determine whether lncRNAs implicated in the odontoblastic differentiation of hDPCs. According to the microarray expression profiles, the expression levels of 1106 lncRNAs were significantly changed by more than 2.0-fold; 617 of these were upregulated, and 489 were downregulated. Among those lncRNAs, MALAT1, MIR31HG, H19, and WNT2 were selected for further identification, based on reports that concentrated on lncRNAs regulating biological mineralization [20, 21]. qRT-PCR results were most consistent with the lncRNA microarray results that H19 expression increased remarkably at day 7 after mineralization induction, so H19 was chosen as a candidate lncRNA.

We subsequently evaluated the functional effects of H19 by transfecting lentiviruses or plasmids into hDPSCs. Overexpression of H19 significantly promoted the odontoblastic differentiation of hDPSCs, whereas lentivirus-mediated silencing of H19 had the opposite effects on hDPSCs. In addition, an ectopic odontogenesis nude mice model was used to further confirm the role of H19 in hDPSCs in vivo. Both micro-CT analysis and histological examination revealed significantly increased levels of dentin-like structure formation and greater abundance of odontogenic-specific markers DMP-1 in H19-overexpression hDPSC-loaded group, which were consistent with the in vitro experiments. Our results were consistent with Zeng et al. [22]. H19 plays a critical role in the differentiation of many vital organs and plays pivotal roles in the differentiation of mesenchymal stem cells [23–26]. Keniry et al. [27] found that H19 can enhance the expression level of RUNX2 to promote bone regeneration. Zhou et al. [28] revealed that H19 can help in bone tissue reparation by inhibiting p53. As a biomineralization promoter, H19 can promote the osteogenic differentiation of mesenchymal stem cells (MSCs) through the Notch signaling pathway [25]. One recent study revealed that H19 enhanced the osteo/dentinogenesis of SCAPs in vivo via the miR-141/SPAG9 pathway [29]. However, the regulatory effect of H19 in odontoblastic differentiation and underlying molecular mechanisms were not entirely identified.

Numerous studies have demonstrated that lncRNA mainly act as miRNA sponges to play various regulatory roles [30–32]. Here, we hypothesized that H19 could promote the odontoblastic differentiation of hDPSCs via sponging miRNAs. Three database, including Starbase, DIANA-LncBase, and RegRNA, predicted six mineralization-related miRNAs (miR-17-5p, miR-93-5p, miR-103a-3p, miR-106b-5p, miR-140-5p, and miR-148-5p) that may have potential binding sites with H19. The expression levels of these candidate miRNAs were evaluated after 14 days of induction. Compared with the non-induction group, the expression levels of miR-140-5p were remarkably decreased in the induction group. Dual-luciferase assay was performed to affirm the direct binding between H19 and miR-140-5p. Later results confirmed the inhibitory effect of miR-140-5p on odontoblastic differentiation by transfecting miRNA mimics into hDPSCs. The expression levels of odontoblast-related genes and matrix mineralization levels were obviously decreased by overexpression of miR-140-5p, compared with miR-NC mimics.

Zheng et. al. [33] revealed that lncRNA MEG3 promoted the osteogenesis of hADSCs via sponging miR-140-5p, indicating the inhibitory role of miR-140-5p in osteogenic differentiation. Our rescue experiments indicated that H19 could partially abrogate the inhibitory effects on odontoblastic differentiation induced by miR-140-5p in vitro which implied that H19 is involved in the ceRNA regulatory network and acts as a miR-140-5p sponge. This is the first report about the regulation of H19/miR-140-5p axis in odontogenic differentiation of hDPSCs. These results showed that H19 promoted the odontoblastic differentiation of hDPSCs by interacting with miR-140-5p. Our findings provided an explanation for the mechanism role of H19 in odontogenesis.

Lu et. al. [34] reported that miR-140-5p regulated the odontoblastic differentiation of DPSCs via targeting Wnt1/β-catenin signaling. Sun et al. [35] found that miR-140-5p-mediated regulation of the proliferation and differentiation of human dental pulp stem cells occurs through the lipopolysaccharide/toll-like receptor 4 signaling pathway. However, the downstream target genes regulated by H19/mir-140-5p axis remain unclear. To reveal the downstream molecular mechanism of H19/miR-140-5p pathway regulating the odontoblastic differentiation of hDPSCs, miRDB and TargetScan were applied to search for potential targets. BMP-2 and FGF9 were predicted as the candidate target genes since the 3′-UTR region of BMP-2 and FGF9 contained potential binding sites with miR-140-5p respectively. Dual-luciferase reporter assays were conducted to further confirm the interaction. Overexpression of miR-140-5p significantly reduced the luciferase activity in BMP-2-wild and FGF9-wild groups, compared with HEK293T cells co-transfected with miR-140-5p mimics and the BMP-2-mut/FGF9-mut 3′UTR vector groups separately. Hwang et al. [36] found that miR-140-5p suppressed BMP2-mediated osteogenesis in undifferentiated human mesenchymal stem cells. Rothman et al. [37] revealed that miR-140-5p mimic altered BMP signaling in pulmonary arterial smooth muscle cells. Much of the literature acknowledges that FGF9 is essential for osteogenesis [38–40], but the relationship between FGF9 and H19 has not been described. Overexpression of H19 increased the expression level of BMP-2 and FGF9, whereas overexpression of miR-140-5p decreased BMP-2/FGF9 mRNA and protein expression. The expression level of BMP-2/FGF9 was partly increased in hDPSCs co-transfected with H19 and miR-140-5p mimics compared to hDPSCs transfected with miR-140-5p mimics alone. Therefore, our results indicated that H19 regulated BMP-2/FGF9 expression by inhibiting the effect of miR-140-5p.

In this study, the biological role of BMP-2 and FGF9 in regulating the odontogenic differentiation of hDPSCs has been verified. hDPSCs cultured in osteogenic induction medium enriched with BMP-2 or FGF9 separately were demonstrated to encourage osteogenic differentiation compared to the normal osteogenic induction group. Furthermore, the results showed that the odontogenic capacity of BMP-2 was better than that of FGF9. It is well recognized that BMP-2 is a critical growth factor and important biomarker involved in osteo/odontoblastic differentiation and bone formation [41–43]. Our results revealed the similar trend that BMP-2 offered a strong signal for differentiation and mineralization of hDPSCs, but was different from Lu et al. [44] which indicated that exogenous FGF9 suppress osteogenic differentiation. This difference may be due to the different stage of hDPSCs and the compensation effect between BMP-2 and FGF9.

In summary, our results indicated that H19/miR-140-5p pathway promoted the odontoblastic differentiation of hDPSCs partially through the regulation of BMP-2 and FGF9.

## Conclusion

In summary, our results demonstrated that H19 was upregulated in hDPSCs during odontogenic induction. Mechanistically, H19 could significantly promote odontogenic differentiation of hDPSCs through competitively binding to miR-140-5p and subsequently reduce the inhibitory effects of miR-140-5p on BMP-2 and FGF9.

## Supplementary information

**Additional file 1: Table S1.** The sequences of Primers used in this study.

## Data Availability

The datasets used or analyzed during the current study are available from the corresponding author on reasonable request.

## Abbreviations

hDPSCs: Human dental pulp stem cells
lncRNA: Long non-coding RNA
cDNA: Complementary DNA
qRT-PCR: Quantitative real-time polymerase chain reaction
DSPP: Dentin sialophosphoprotein
DMP-1: Dental matrix protein-1
ALP: Alkaline phosphatase
RUNX2: Runt-related transcription factor 2
BMP-2: Bone morphogenetic protein 2
FGF9: Fibroblast growth factor 9
hBMSCs: Human bone mesenchymal stem cells
MSCs: Mesenchymal stem cells
ceRNA: Competing endogenous RNA
miRNA: MicroRNA
3′UTR: 3′ untranslated region
NC: Negative control
