# Supplementary material for: LncRNA H19 promotes odontoblastic differentiation of human dental pulp stem cells by regulating miR-140-5p and BMP-2/FGF9
Source: Stem Cell Res Ther. 2020 May 27;11:202. doi: 10.1186/s13287-020-01698-4 (PMC7251819; doi:10.1186/s13287-020-01698-4)
Supplement: Supplementary file 1 — Additional file 1: Table S1. The sequences of Primers used in this study. [file 13287_2020_1698_MOESM1_ESM.doc]

LncRNA H19 Promotes Odontoblastic Differentiation of Human Dental Pulp Stem Cells by Regulating miR-140-5p and BMP-2/FGF9

Jialin Zhong[1#], Xinran Tu[1#], Yuanyuan Kong[1], Liyang Guo[1], Baishun Li[1], Wenchao Zhong[1], Ying Cheng[2], Yiguo Jiang[2*] and Qianzhou Jiang[1*].

1 Key Laboratory of Oral Medicine, Guangzhou Institute of Oral Disease, Stomatology Hospital of Guangzhou Medical University, Huangsha Avenue 39, Guangzhou 510000, P. R. China.

2 State Key Laboratory of Respiratory Disease, Institute for Chemical Carcinogenesis, Guangzhou Medical University, Xinzao, Panyu District, Guangzhou 511436, P. R. China.

Jialin Zhong and Xinran Tu contributed equally to this work.

[*]Corresponding authors.

Table 1. The sequences of Primers used in this study

Gene	Forward primer sequences	Reverse primer sequences
MALAT1	GTTTGGTCTTGGGGTTTG	ACTGCTCTGGGTCTGCTT
MIR31HG	CGTAAAGTGTGGAGTTGGTC	CCAGGAGAAGGTGGAAGT
WNT2	CCTTTGGGTTCTTTCTGG	CGTCTCCACAACTCCAAC
H19	CACCACCTCCCTCTTCTT	CGTCTCCACAACTCCAAC
DSPP	CTATGGAAGGATGGAGGAAAG	ACTGGGTAGGAATGTGGATGA
DMP-1	GCCACCCCACTTGGTCTACTA	TCACCCTCTGAAGGTTTGCTG
ALP	TTCCAACTGCCTCTAACTCCT	CTCTGTCCAAGGTAAGTGCTG
GAPDH	AAGAAGGTGGTGAAGCAGG	GTCAAAGGTGGAGGAGTGG
U6	CGCTTCGGCAGCACATATAC	TTCACGAATTTGCGTGTCAT
Runx2	ACCCAGAAGGCACAGACAGAAG	AGGAATGCGCCCTAAATCACT
miR-140-5p	GCGCCAGTGGTTTTACCCTA	
Uni		GTGCAGGGTCCGAGGT

Supplementary Figure

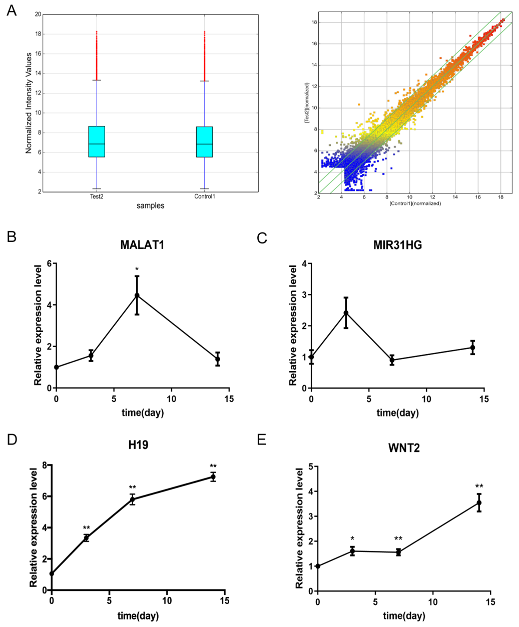

Supplementary Figure 1 The expression levels of lncRNAs in hDPSCs after induction for 14 days.

A. Differentially expressed lncRNAs in the control and test groups (control: hDPSCs cultured with normal medium, test: hDPSCs cultured with odontoblastic differentiation-inducing medium).

B-E. The expression levels of four selected lncRNAs during odontoblastic differentiation. The data are presented as the mean ± SD of three experiments. *$^*P<0.05$, $^{**}P<0.01$.*
